# Supplementary material for: The haplolethal gene wupA of Drosophila exhibits potential as a target for an X-poisoning gene drive
Source: G3 (Bethesda). 2024 Feb 2;14(4):jkae025. doi: 10.1093/g3journal/jkae025 (PMC10989859; doi:10.1093/g3journal/jkae025)
Supplement: jkae025_Supplementary_Data [file jkae025_supplementary_data.zip › Figure_S1_G3-2024-404841.pdf]

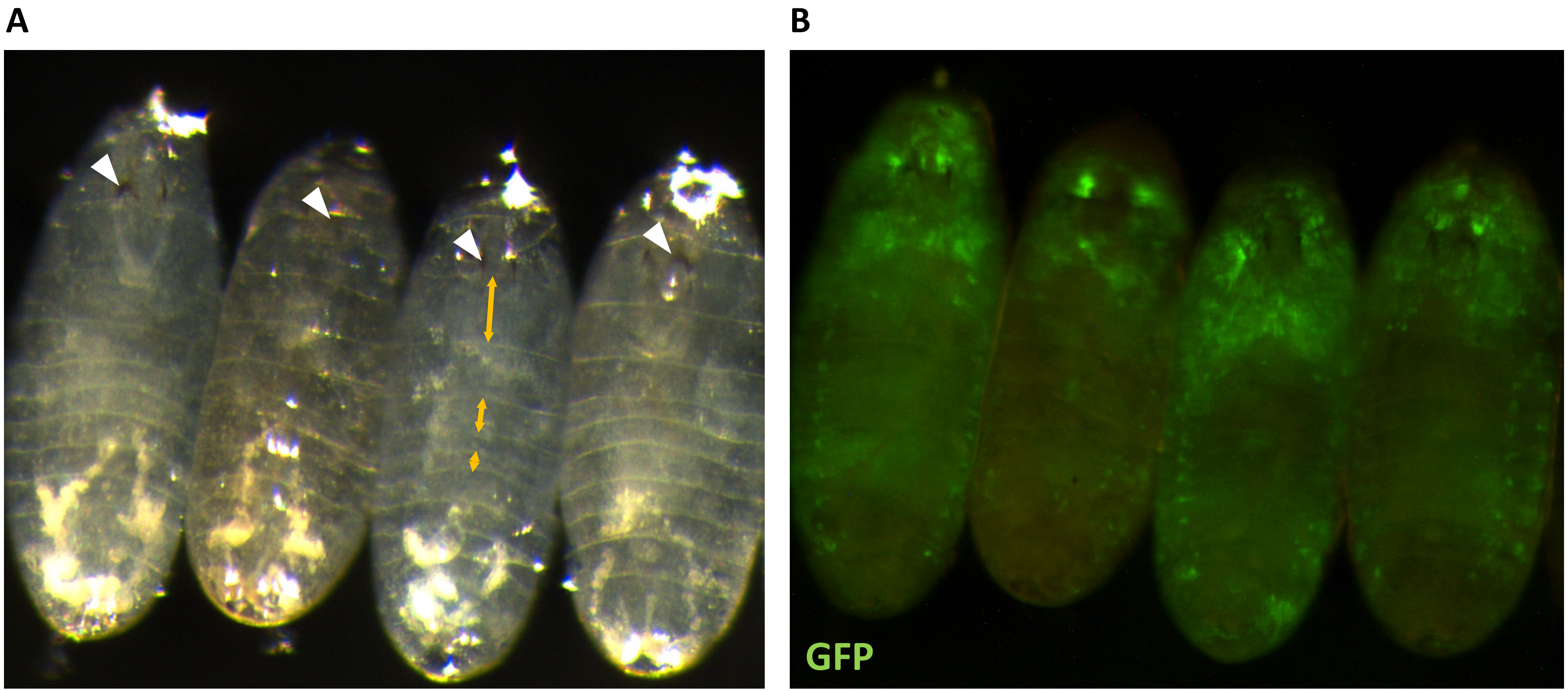

Supplementary Figure S1. Dorsal view of four representative embryos (that are putatively heterozygotes for wupA mutations) illuminated under stereomicroscope. Embryos are ~48 hours old and presumed deceased. (A) Mouthparts (white arrowheads) and unevenly spaced segmental furrows (yellow arrows). (B) Fluorescent stereomicroscopy. GFP indicates that they are female.
