# Supplementary material for: The haplolethal gene wupA of Drosophila exhibits potential as a target for an X-poisoning gene drive
Source: G3 (Bethesda). 2024 Feb 2;14(4):jkae025. doi: 10.1093/g3journal/jkae025 (PMC10989859; doi:10.1093/g3journal/jkae025)
Supplement: jkae025_Supplementary_Data [file jkae025_supplementary_data.zip › Figure_S2_G3-2024-404841.pdf]

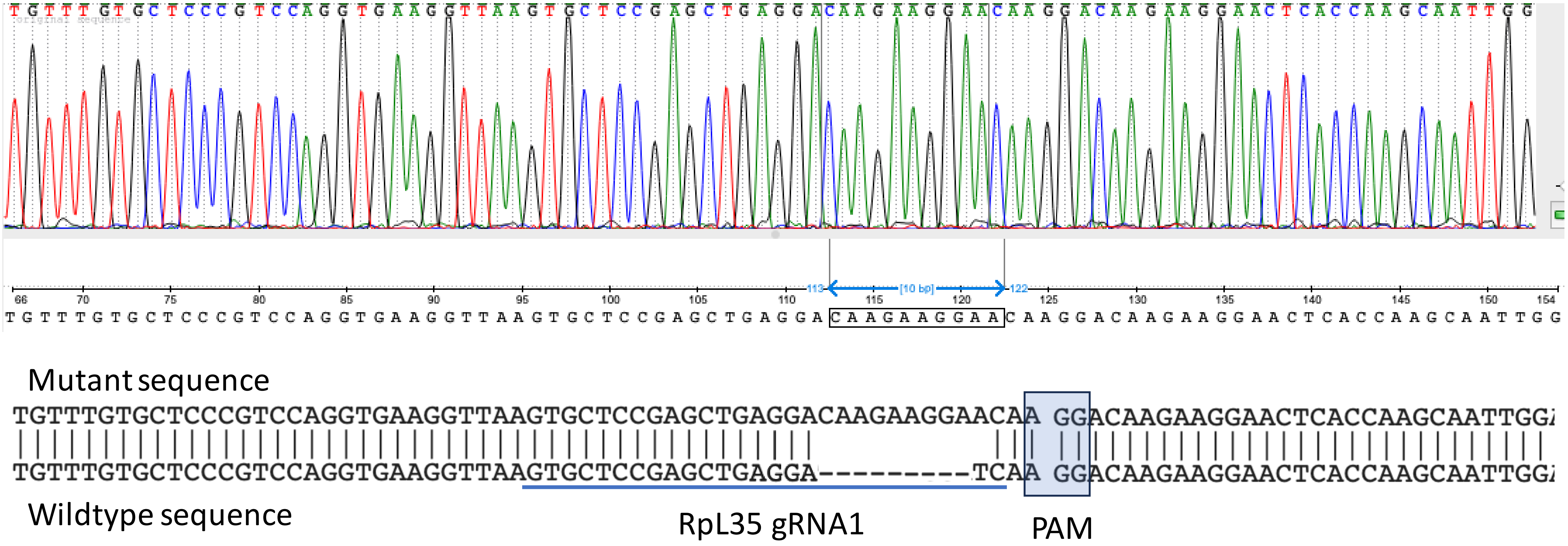
[10 bp]
TGTTTGTGCTCCCGTCCAGGTGAAGGTTAAGTGCTCCGAGCTGAGGACAAGAAGGAACAAGGACAAGAAGGAACTCACCAAGCAATTGG
Mutant sequence
TGTTTGTGCTCCCGTCCAGGTGAAGGTTAAGTGCTCCGAGCTGAGGACAAGAAGGAACAA GGACAAGAAGGAACTCACCAAGCAATTGG
TGTTTGTGCTCCCGTCCAGGTGAAGGTTAAGTGCTCCGAGCTGAGGA---------TCAA GGACAAGAAGGAACTCACCAAGCAATTGG
Wildtype sequence
RpL35 gRNA1
PAM
