# Supplementary material for: The haplolethal gene wupA of Drosophila exhibits potential as a target for an X-poisoning gene drive
Source: G3 (Bethesda). 2024 Feb 2;14(4):jkae025. doi: 10.1093/g3journal/jkae025 (PMC10989859; doi:10.1093/g3journal/jkae025)
Supplement: jkae025_Supplementary_Data [file jkae025_supplementary_data.zip › Figure_S4_G3-2024-404841.pdf]

```
CLUSTAL O(1.2.4) multiple sequence alignment

sgRNA2                               AEVRKME
Dmelanogaster      MADDEAKKAKQAEIERKRAEVRKRMEEASKAKKAKKGFMTPERKKKLRLLLRKKAAEELK         60
Dsuzukii           MADDEAKKAKQAEIERKRAEVRKRMEEASKAKKAKKGFMTPERKKKLRLLLRKKAAEELK         60
Amarshallii        MADDEAKKAKQAEIERKRAEVRKRMEEASKAKKAKKGFMTPERKKKLRLLLRKKAAEELK         60
Astephensi         MADDEAKKAKQAEIERKRAEVRKRMEEASKAKKAKKGFMTPERKKKLRLLLRKKAAEELK         60
Amaculipalpis      MADDEAKKAKQAEIERKRAEVRKRMEEASKAKKAKKGFMTPERKKKLRLLLRKKAAEELK         60
Agambiae           MADDEAKKAKQAEIERKRAEVRKRMEEASKAKKAKKGFMTPERKKKLRLLLRKKAAEELK         60
Acoluzzii          MADDEAKKAKQAEIERKRAEVRKRMEEASKAKKAKKGFMTPERKKKLRLLLRKKAAEELK         60
                   ************************************************************

sgRNA3                        RIIEERC
Dmelanogaster      KEQERKAAERRRIIEERCGSPRNLSDASEDTLKSLIKQHYDRINKLEDQKYDLEYVVKRK         120
Dsuzukii           KEQERKAAERRRIIEERCGSPRNLSDASEDTLKSLIKQHYDRINKLEDQKYDLEYVVKRK         120
Amarshallii        KEQERKAAERRRIIEERCGKPKNIEEANEDQARKILRDYHQRINNLEEEKYDLEYVVKRK         120
Astephensi         KEQERKAAERRRIIEERCGKPKNIEEANEDQARKILRDYHQRINSLEEEKYDLEYVVKRK         120
Amaculipalpis      KEQERKAAERRRIIEERCGKPKNIEDANEDQARKILRDYHQRINSLEEEKYDLEYVVKRK         120
Agambiae           KEQERKAAERRRIIEERCGKPKNVEDANEDQARKILRDYHQRINSLEEEKYDLEYVVKRK         120
Acoluzzii          KEQERKAAERRRIIEERCGKPKNVEDANEDQARKILRDYHQRINSLEEEKYDLEYVVKRK         120
                   *******************.*:*:..:*.**  :.::::::***.**::***********

Dmelanogaster      DVEINDLNAQVNDLRGKFVKPALKKVSKYENKFAKLQKKAAEFNFRNQLKVVKKKEFTLE         180
Dsuzukii           DVEINDLNAQVNDLRGKFVKPALKKVSKYENKFAKLQKKAAEFNFRNQLKVVKKKEFTLE         180
Amarshallii        DMEISDLNAQVNDLRGKFVKPTLKKVSKYENKFAKLQKKAAEFNFRNQLKVVKKKEFTLE         180
Astephensi         DMEISDLNAQVNDLRGKFVKPTLKKVSKYENKFAKLQKKAAEFNFRNQLKVVKKKEFTLE         180
Amaculipalpis      DMEISDLNAQVNDLRGKFVKPTLKKVSKYENKFAKLQKKAAEFNFRNQLKVVKKKEFTLE         180
Agambiae           DMEISDLNAQVNDLRGKFVKPTLKKVSKYENKFAKLQKKAAEFNFRNQLKVVKKKEFTLE         180
Acoluzzii          DMEISDLNAQVNDLRGKFVKPTLKKVSKYENKFAKLQKKAAEFNFRNQLKVVKKKEFTLE         180
                   *:**.****************:**************************************

Dmelanogaster      EEEKEKKPDWSKGKPGDAKVKEEVEA----EA        208
Dsuzukii           EEEKEKKPDWSKGKPGDAKVKEEVEA----EA        208
Amarshallii        EEDKEKKPDWSKKG--DK-VKEEAAEAVEAEA        209
Astephensi         EEDKEKKPDWSKKG--DSKVKEEAAEAVEAES        210
Amaculipalpis      EEDKEKKPDWSKKG--DSKVKEEAAEAVEAEA        210
Agambiae           EEDKEKKPDWSKKG--DSKVKEEAAEAVEAEA        210
Acoluzzii          EEDKEKKPDWSKKG--DSKVKEEAAEAVEAEA        210
                   **:*********    *  ****.      *:
```

```
Percent Identity  Matrix - created by Clustal2.1

     1: Dmelanogaster  100.00
     2: Dsuzukii       100.00  100.00
     3: Amarshallii     85.37   85.37  100.00
     4: Astephensi      84.95   84.95   98.56  100.00
     5: Amaculipalpis   85.92   85.92   98.56   99.05  100.00
     6: Agambiae        85.92   85.92   98.09   98.57   99.52  100.00
     7: Acoluzzii       85.92   85.92   98.09   98.57   99.52  100.00  100.00
```

**Species**	***wupA* NCBI Reference Sequence**
Drosophila melanogaster	NP_728141.1 (Isoform A)
Drosophila suzukii	XM_017083743.2 (Transcript X7)
Anopheles marshallii	XM_053811567.1 (Transcript X4)
Anopheles stephensi	XM_036057764.1 (Transcript X6)
Anopheles maculipalpis	XM_050216580.1 (Transcript X2)
Anopheles gambiae	XM_003437142.2 (Transcript X5)
Anopheles coluzzii	XM_040364350.2 (Transcript X2)
